# Impact of Salinity Gradients on Seed Germination, Establishment, and Growth of Two Dominant Mangrove Species Along the Red Sea Coastline

**DOI:** 10.3390/plants13243471

**Published:** 2024-12-11

**Authors:** Fahad Kimera, Basma Sobhi, Mostafa Omara, Hani Sewilam

**Affiliations:** 1 Center for Applied Research on the Environment and Sustainability (CARES), School of Science and Engineering, The American University in Cairo, AUC Avenue, P.O. Box 74, New Cairo 11835, Egypt; basma.khalil@aucegypt.edu (B.S.); mostafa_metwally@aucegypt.edu (M.O.); 2 Department of Engineering Hydrology, Faculty of Civil Engineering, RWTH Aachen University, 52074 Aachen, Germany

**Keywords:** mangroves, salinity, *Avicennia marina*, *Rhizophora mucronata*

## Abstract

Background: Mangroves are one of the key nature-based solutions that mitigate climate change impacts. Even though they are halophytic in nature, seedlings are vulnerable to high salinity for their establishment. This study investigated the effects of different salinities on seedling growth and mineral element composition of two dominant species (*Avicennia marina* and *Rhizophora mucronata*). Methods: The study followed a randomized complete block design, i.e., main treatments (growing environment in greenhouse (GH) or net house (NH)) and four sub-treatments under 21 replicates, i.e., irrigation with 100% freshwater (0.4%o—T1), 100% saline water (35%o—T2), 50% saline water and 50% freshwater (18%o—T3), and brine water (60%o—T4). Results: Results revealed that *A. marina* seeds can optimally germinate and survive well reaching 80% in NH under T1. However, T2 and T4 seedlings had the lowest survival. Mineral element analysis showed that *A. marina* grown under NH recorded higher levels of Ca, Mg, and K which increased with increasing levels of salinity. The opposite was true with Na levels. *R. mucronata* on the other hand, recorded completely opposite findings with T1 seedlings reaching 95% in the greenhouse while T3 reached almost 60%. Conclusions: It can be concluded that mangrove species can optimally germinate and grow in both freshwater and 50% saline water, but growth reduction occurs with seawater and complete growth inhibition with brine water.

## 1. Introduction

**Figure 1:**
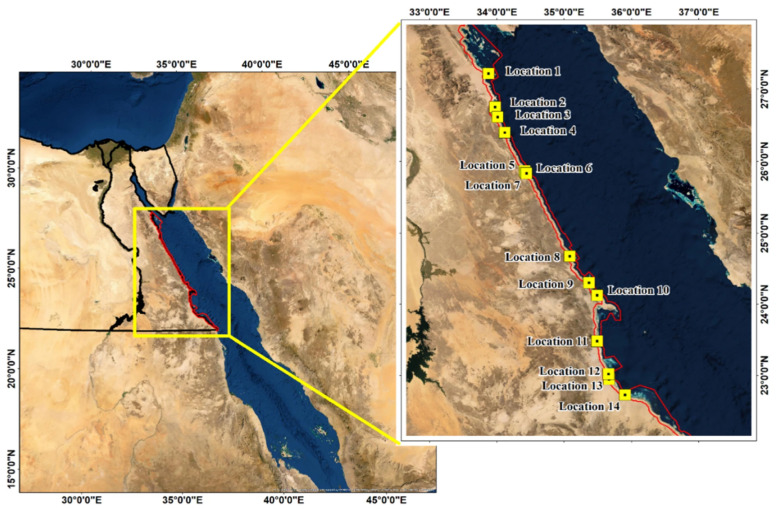
Mangrove locations and distribution along the Egyptian Red Sea coastline, adopted from [6].

Mangroves refer to a broad collection of woody plant species growing in tropical and sub-tropical marine intertidal zones [1]. According to [2], mangrove forests can be considered one of the key nature-based solutions that address climate change impacts while providing socio-economic and ecological services. Indigenous people use mangrove trees for a variety of livelihood purposes, including fuel, food baskets, medicinal goods, construction materials, and fishing gear [3]. However, climate change may lead to a 10–15% loss of mangroves globally [4]. Due to deforestation alone, mangroves are also disappearing at the rate of 1–2% per year despite their significant importance. Globally, there are 84 mangrove plant species distributed across the continent with 24 genera and 16 families. Amongst the total species, only 70 are considered true mangroves dominated by both *Avicennia* and *Rhizophora* [5]. In Egypt, there are two dominant mangrove species distributed along the Red Sea coastline. Figure 1 shows the total area of 512 ha of coastal land occupied by the two mangrove species along the Egyptian Red Sea coastline [6].

*Avicennia marina* (gray mangrove) belongs to the *Avicenniaceae* family [7]. The plant reaches a height of 10 m with aerial roots that can reach 20 cm in height with a diameter of approximately 1 cm [8]. It is the most common mangrove species in the Indo-Western Pacific region, South and Southeast Asia, the Middle East, and along the African coast [5,9]. It is highly resistant to adverse environmental conditions and significantly adapts to high saline conditions, drought, wide temperature ranges, tidal inundation, rain, and frost frequency [10,11,12,13]. *Rhizophora mucronata* (Red mangrove) belongs to the Rhizophoraceae family distributed along the coasts of tropical and subtropical regions [14]. Trees are typically 15–25 m high, with yellowish leaf stipules and tiny black spots on the underside of the leaves.

Mangrove characteristics can be affected by temperature, coastal types, sea currents and land barriers, wave movements and sediment supply, river catchment runoff and sediment yields, tidal range, and flood frequency [15,16]. Because mangroves are vulnerable to freezing and chilling conditions, they are found mainly in the tropics and subtropics. Mangroves have a specified threshold limit of at least 20 °C mean winter sea surface temperature [15,17]. However, black mangroves can tolerate low temperatures and can recover from freeze damage [18]; due to the decreasing frequency and intensity of freeze events induced by climate change, mangroves are now increasingly spreading into temperate zones on many continents [19].

Decreased rainfall and humidity lead to reduced mangrove diversity, photosynthesis, productivity, and growth. In areas of low precipitation, high evaporation, and higher salinity, it can lead to the stunting of mangroves or replacement by hypersaline sand flats. Such regions may experience widespread mangrove mortality during the drought period [20].

Mangroves can be regenerated in two possible ways, i.e., artificial and natural regeneration. The latter method uses naturally occurring propagules or seeds of mangroves as the source for regeneration. Artificial regeneration means collecting ripe seeds or propagules and planting them directly into the site or raising seedlings under nursery conditions and then transplanting them to the field. The selection of mangroves to be planted is generally determined by three factors: the mangrove species occurring naturally in the locality of the afforestation site and the availability of seeds or propagules.

Saline conditions play a role in accelerating the growth of mangroves; however, there is a difference between species in terms of the range of salinities they can grow under and sustain growth. Some authors have previously reported that the required salinity for optimum growth starts from 10% to 50% seawater [17,21,22,23].

Depending on the available literature, few or no studies have investigated the salinity effects on seedling growth and the establishment of these two dominant species on the Red Sea under different growing environments. The current experiment studies the effects of different salinity levels on seed germination, seedling establishment, and seedling growth of two dominant mangrove species (*Avicennia marina* and *Rhizophora* mucronata) grown in two different environments (controlled cooling greenhouse and net greenhouse).

## 2. Results and Discussions

### 2.1. Seedling Germination, Growth and Survival

**Figure 2:**
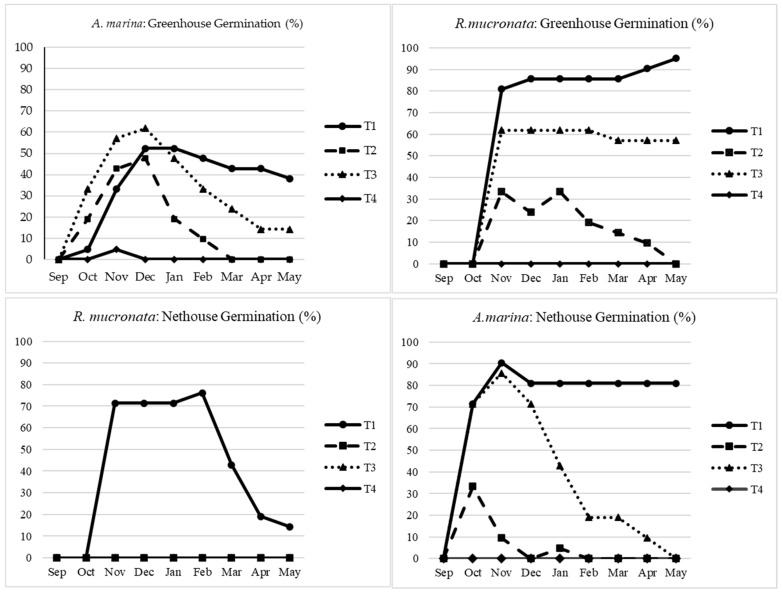
Germination and survival rates of two mangrove species were assessed eight months after sowing, encompassing the entire seedling development period. The absence of some lines in the Figure means that the plants never germinated, and hence, no survival was recorded. T1: freshwater, T2: seawater, T3: mixed water, T4: brine.

Mangroves play a very significant role in environmental purification. Sea water salt concentration is vital for the establishment and distribution of mangrove species globally. Even though mangroves are amongst the commonly known halophytic trees, extreme salt concentration in their growth environment can significantly affect their growth and establishment. Generally, our study revealed that A. marina seeds can optimally germinate and grow better in net house conditions rather than in a cooling greenhouse as seen in Figure 2. In the case of the net house, the T1 seedlings recorded a survival rate of 80% as compared to 38% for the greenhouse. T3, however, recorded lower survival rates (19 and 14%) for net house and greenhouse, respectively as compared to T1. Even though some seeds from T2 and T4 emerged in the first few months, the seedlings did not survive and almost all died by the end of the experimental period; statistically, T2 and T4 were not significantly different at *p* < 0.05.

On the other hand, as can be observed in Figure 2, *R. mucronata* recorded totally opposite findings regarding the survival rate. The T1 seedlings reached 95% in the greenhouse versus 19% for the net house, these differences were significantly different at *p* < 0.05. While T3 only survived inside the greenhouse reaching 57%. There was no significant difference between T1 and T3 under the greenhouse setup; however, there was a significant statistical difference between T1 and all other treatments in the net house (*p* < 0.05). These survival differences for the different mangrove species could be attributed to their genetic differences, local weather patterns in most particularly the temperature differences between the greenhouse and the net house. Mangrove plants thrive in tropical and subtropical climates, where their growth is optimally influenced by warm weather conditions. However, during winter months, exposure to cold temperatures may adversely affect the growth of seedlings, particularly when they are cultivated outside a greenhouse environment. In a previous study, ref. [22] investigated the germination of *A. marina* and *R. mucronata* in saline water; they reported that 100% seawater inhibited mangrove germination and growth in both species under uncontrolled greenhouse establishment.

Furthermore, refs. [1,24] reported that mangrove seedlings require moderate salinity (50% seawater) to germinate but as they grow and establish, they tend to be highly saline tolerant. Similar to our results, so many other authors have reported mangrove seed germination optimum at 50% seawater dilution [22,25,26]. Additionally, the authors reported that mangrove seedling germination is more efficient with freshwater, which greatly conforms to our results too. Saline water reduces and impedes the growth of plants due to low water potential, ion toxicities, and nutrient deficiencies. In other contradicting findings, according to many growth studies, the optimal salinity for *Avicennia marina* growth varies between 10% and 90% of seawater; however, for yet unknown reasons, poor freshwater growth has been previously observed [27]. According to the same source, *Avicennia marina* seedlings did not grow in 0–5% seawater, but maximum growth occurred with a 50–75% increase in seawater.

### 2.2. Seedling Establishment and Height

Since *Avicennia marina* was sown from small seeds of uniform size, the experiment assessed the final seedling height at the end of the experimental period for all treatments. T1 expressed the highest average seedling height reaching almost 22 cm in the greenhouse against 7.2 cm for the net house. T1 was significantly different between GH and NH across all treatments. Whereas T3 with 8.4 cm in the greenhouse versus zero height for the net house, these differences were also found significantly different at *p* < 0.05. T2 and T4 did not record any final plant height since the plants recorded zero survival rates by the end of the experimental time.

For the case of *Rhizophora*, the initial size of the seeds is quite different from *Avicennia* since the genus *Rhizophora* seeds are of durian type, i.e., have an extended hypocotyl. So, the initial average height of the seeds was (31.2 ± 2.92) cm.

**Figure 3:**
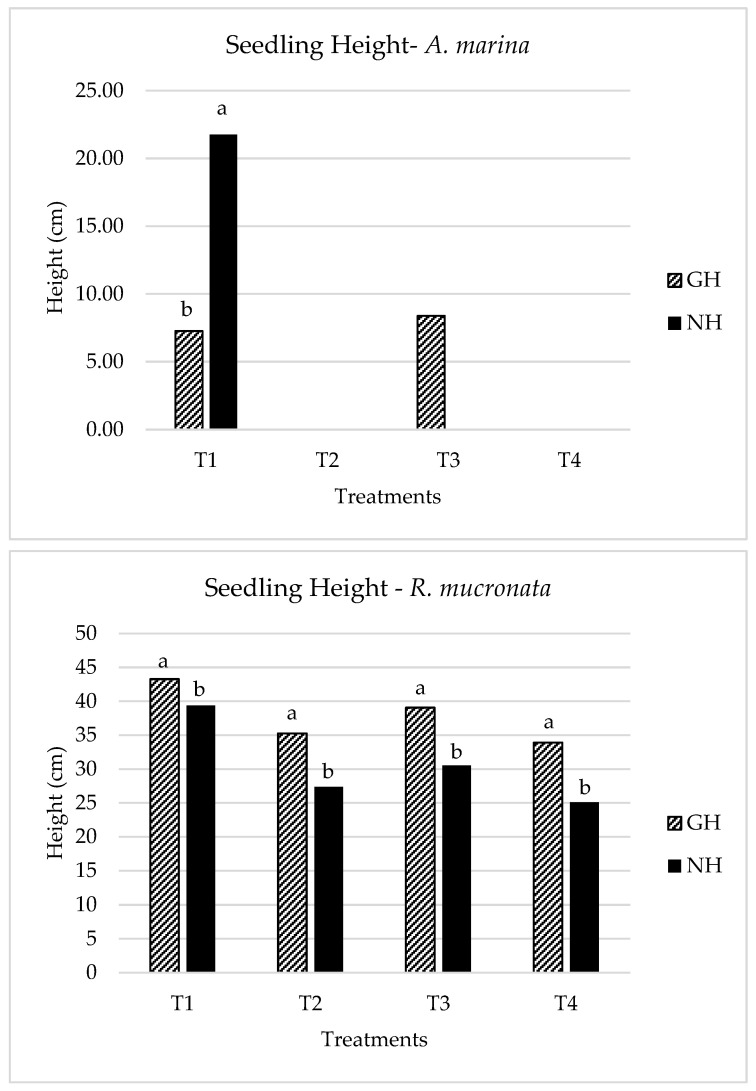
Final seedling height for both species at the end of the study period. GH: greenhouse, NH: net house, T1: freshwater, T2: seawater, T3: mixed water, T4: brine. Bars having different small letters above them under the same treatments mean a significant difference between them at *p* < 0.05.

However, after eight months of experimentation, T1 in the greenhouse recorded a significantly higher final seedling height reaching 43.2 cm with a height difference of 4 cm relative to the highest in net house. T3 followed closely with 39 cm height for the case of the greenhouse and finally, T2 and T4 followed, respectively, with decreased seed heights instead. While comparing the final plant height of *R. mucronata* with the Initial seed height for T2 and T4, the seedling height decreased for both treatments since the seeds instead of growing, stunted and shrunk as can be observed in Figure 3. Ref. [22] reported an increased plant height at 50% seawater irrespective of the species, which decreased with an increased salinity level. They also elaborated on the potential of *A. marina* accumulating more ions in its tissues as compared to *R. mucronata*. Generally, the *Avicennia* seedling heights were highest in net house experimentation while the *Rhizophora* seedlings recorded the highest seedling height in the greenhouse settings.

### 2.3. Seedling Stem Diameter and Leaf Parameters

The stem diameter for *Avicennia* increased throughout the growing time with a maximum of 3.2 cm for T1 in the net house. Treatments T2 and T4 did not record any values by the end of the experiment since the plants did not survive, as shown in Table 1.

**Figure 4:**
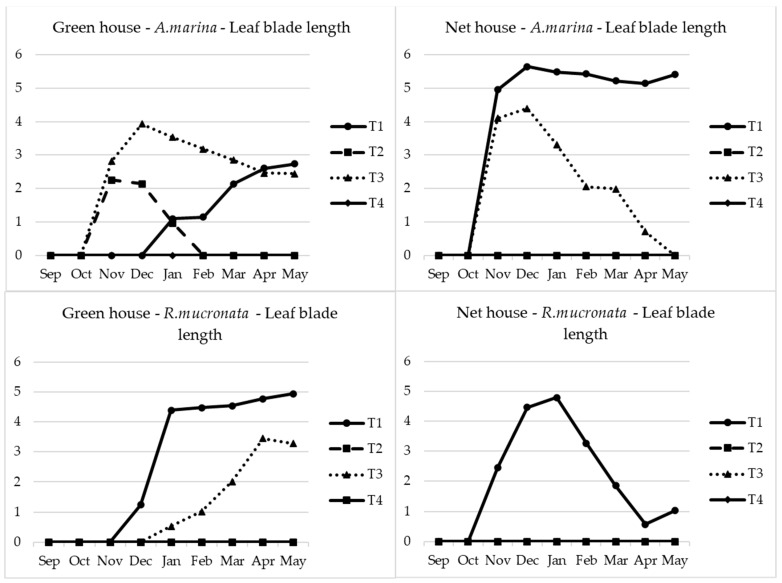
Leaf blade length of both species. The absence of some lines in the Figure means that the plants never germinated, and hence, no leaf parameters were recorded: T1: freshwater, T2: seawater, T3: mixed water, T4: brine.

**Figure 5:**
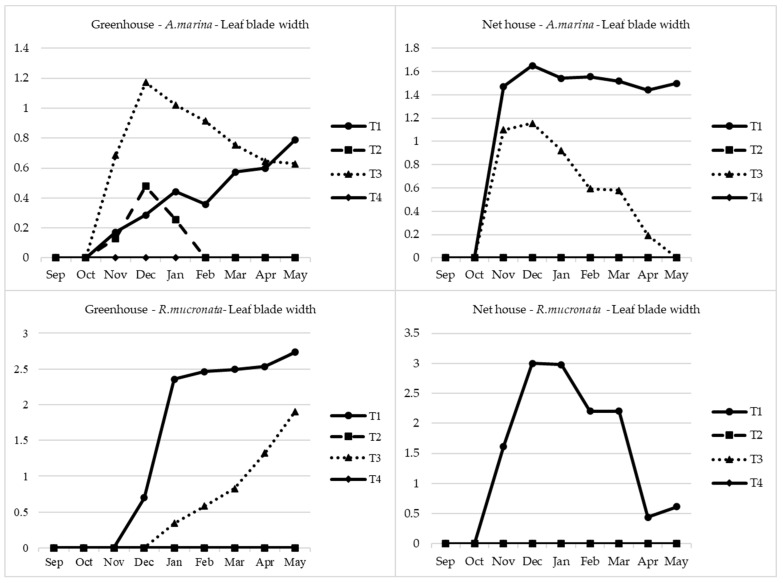
Leaf blade width of both species. The absence of some lines in the Figure means that the plants never germinated, and hence, no leaf parameters were recorded: T1: freshwater, T2: seawater, T3: mixed water, T4: brine.

For the *Rhizophora*, seedlings generally decreased or remained constant throughout the experimentation period except for the T1 seedlings. It was the same observation in both the greenhouse and the net house. As was previously observed with the seedling height, the same trend applied to the leaf blade length. It is the net house that gave the highest leaf blade length for *Avicennia* with T1 as the dominant treatment with the maximum significant length of 5.6 cm followed by T3, as can be seen in Figure 4 and Figure 5. On the other hand, in the case of *Rhizophora*, the opposite was observed. T1 and T3 had no significant difference in regard to the leaf blade length in GH irrespective of the species; however, T1 expressed significantly higher leaf blade length in NH irrespective of the species, even though the highest leaf blade length was recorded in the greenhouse reaching 4.5 cm. A similar observation was also recorded with the leaf blade width.

**Figure 6:**
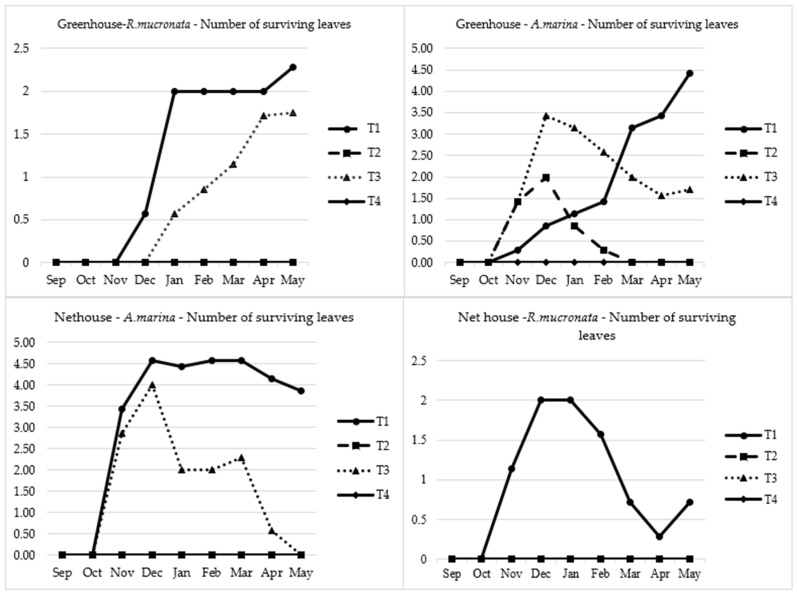
Leaves survivability in both mangrove species. The absence of some lines in the Figure means that the plants never germinated, and hence, no leaf survival was recorded. T1: freshwater, T2: seawater, T3: mixed water, T4: brine.

Regarding the survival of the plants in terms of green canopy for *Avicennia*, the number of surviving leaves per treatment was also assessed. T1 and T3 were established quite well by the beginning of the experiment with a significant number of leaves for the first four months; however, T3 gradually decreased from 3.4 to 1.7 leaves by the end of the experiment in the greenhouse. For the net house, T3 lost all its leaves from four leaves in the middle of the experiment to zero by the end of the experiment period. Contrary, T1 managed to sustain its leaves high in both cases reaching 4.4 leaves and 4.5 leaves in the greenhouse and net house, respectively. This is presented in Figure 6 below. It has been previously reported that mangroves, *Rhizophora* in particular, tend to increase their lignin composition in tissues when exposed to heavy metals [28,29,30]. *Rhizophora* leaf survival was quite tricky; the seedlings narrowly survived, with only 2.3 and less than a leaf for the greenhouse and net house, respectively.

### 2.4. Mineral Element Composition

**Figure 7:**
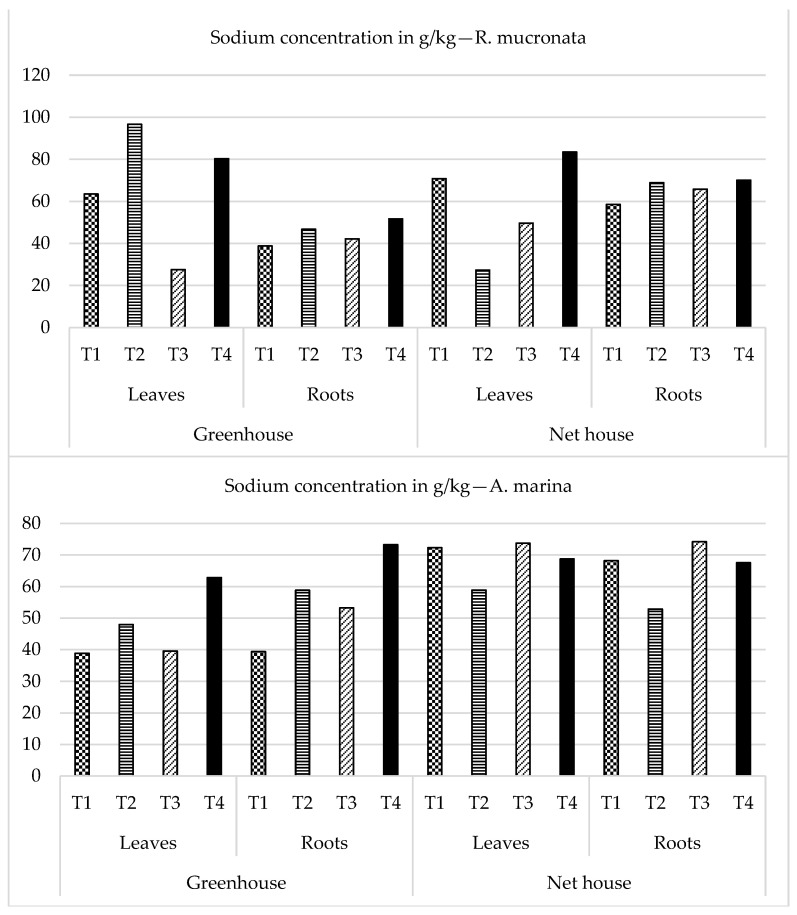
Sodium concentration in different parts of the seedlings at the end of the experimental period. T1: freshwater, T2: seawater, T3: mixed water, T4: brine water.

Mineral analysis for some selected elements was conducted to assess the translocated mineral concentration in different parts of the seedlings as a result of the different water quality treatments under different levels of irrigation salinities. At the end of the experiment, two seedling parts, i.e., the shoot (Leaves and top branches) and the lower part (roots), were collected and analyzed for mineral elements assessment. *A. marina* grown under net house conditions was generally characterized by higher elemental concentration, mainly Na, in both the leaves and roots compared to those grown in a greenhouse. This might have been due to their halophytic nature of translocating saline water into their tissues and being able to regulate and balance their salt levels through different mechanisms. This was the same trend even with *R. mucronata* as seen in Figure 7. *A. marina* grown under a greenhouse generally recorded higher levels of Ca, Mg, and K in both the leaves and roots of T1 and T3 as compared to other saline treatments of T2 and T3. This was not the case for the seedlings in the net house; the levels of Ca, Mg, and K increased with increasing levels of salinity in the water. This is also illustrated in Table 2. There was a similar observed trend with Na too.

On the other hand, the opposite was true with the net house treatments. Na levels in both the shoot and roots decreased with increasing levels of water salinity. An important factor worth mentioning about the mangrove seedlings grown in the net house was the observation of salt crystal accumulation on the leaves of *A*. *marina*. This was more prominent in the seedlings from T2 and T4. Our results were in agreement with those reported by Cheng et al. [23] in the observation of white salt crystals on both sides of *A. marina* leaves. The rate of accumulation of these crystals increased with the order from T3, T2, and T4. Salt excretion appears to be an important adjustment for *Avicennia marina* to survive a high-salt environment.

*R. mucronata* was observed to have very high levels of Ca, Mg, and K in their shoots and leaves compared to the recorded values in *A. marina* irrespective of the study area. The Na concentration in shoots and roots of *R. mucronata* generally increased with increasing levels of irrigation water even though its levels were much higher in net house seedlings. Nevertheless, through its contribution to the osmotic adjustment of developing tissues, salt uptake in these halophytic plants aids in the maintenance of positive pressure potential [31,32]. Salt absorption plays an efficient role in regulating the water potential of seedlings. The study results revealed that higher saline water levels beyond 20‰ can have a significant effect on the germination, survival, and establishment of mangrove seeds which in the long run inhibits the growth of mangrove seedlings. Also, in their field experiment, ref. [1] reported that hypersalinity inhibits growth, reduces leaf area, increases leaf sap osmotic pressure, and increases the leaf area/weight ratio.

In another study, ref. [26] reported that sodium content in the vegetative tissues (leaves and roots) expressed a positive relationship with soil salinity, i.e., an increase in sodium ions was observed with an increase in soil salinity. However, for N, P, K, Ca, and Mg content in tissues had a negative relation with soil salinity; these ions decreased with increasing levels of salinity. In this experiment, older leaves of *A. marina* under net house were observed with some salt crystals from leaf glands. Higher internal osmotic potential in plant tissues growing in saline environments is beneficial for such plants to ensure an osmotic balance. However, if the salt content extremely increases beyond tolerable range, this can impede and inhibit both water and nutrient uptake of the plant.

In the current experiment, it has been revealed that the sodium content in plant tissues increases with an increase in salinity treatments. Generally, for *A. marina*, the Na content was observed more in the roots than in the leaves under greenhouse treatments. It was the opposite finding for the net house treatments, where the Na ions were more concentrated in the leaves than the roots. On the other hand, for *R. mucronata*, Na was more concentrated in the leaves under the greenhouse and its content was somehow comparable under the net house for roots and leaves. Our results suggest and confirm that a higher concentration of Na in plant tissues inhibits K^+^ and Ca^2+^. Similar results were also presented by Patel et al. [33].

As compared to other halophytic plants, mangrove seedlings’ growth is generally enhanced by saline conditions; however, different species respond differently to different salinity ranges. For example, *Sonneratia lanceolata* is a halophytic plant that grows vigorously in freshwater and the opposite is true for other species like *Rhizphora mangle* [27]. In their study, the latter authors in addition to [34] revealed that *A. marina* failed to grow with freshwater; the seedlings only established when exposed to higher salinity levels of 50–75% seawater, which, to an extent, agrees with the results in the present study even though with some contradictions. *A. marina* are known to be highly saline tolerant plants through different adaptation mechanisms such as salt exclusion by roots, salt secretion through leaves and accumulation of high intracellular salt concentrations in leaves and roots [31,32,35,36].

Our results show that *A. marina* is more efficient in dealing with saltwater stress and high climatic temperatures in both arid and semi-arid coastal areas. Germination and growth of these species can be greatly improved if seawater in the coastal areas has been compromised by mixing it with freshwater either with surface runoff after a heavy storm or by human influence. Even though both species are categorized as true salt-tolerant mangroves, several authors have reported that *A. marina* is more tolerant to salinity effects than *R. mucronata* [37].

## 3. Materials and Methods

### 3.1. Experimental Location and Setup

**Figure 8:**
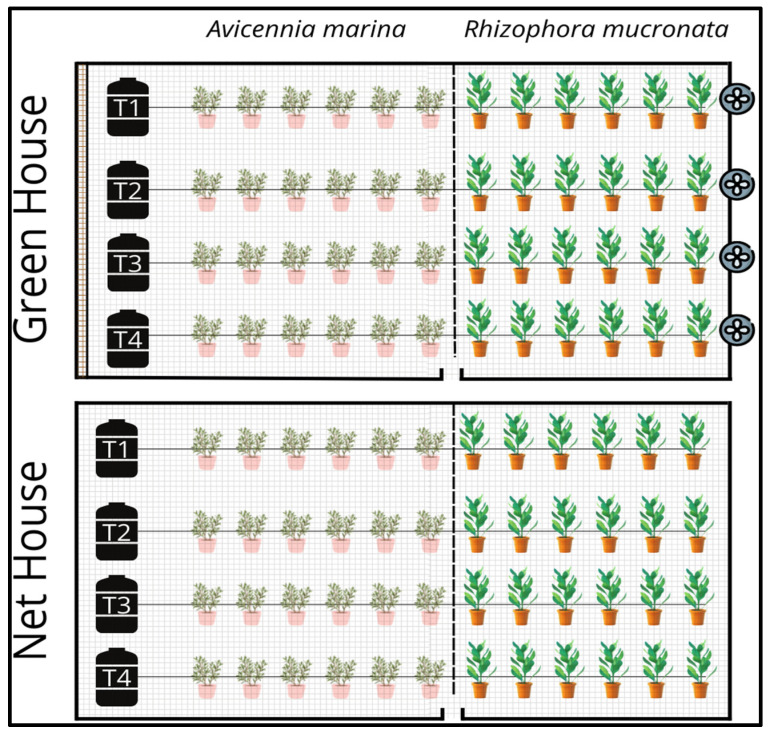
Experimental design and setup.

Seeds of both species *Avicennia marina* and *Rhizophora mucronata* were collected from the mangrove site at Safaga, Red Sea governorate, Egypt (26.62, 34.01) at the end of August 2021. Both seed species were gathered, selected, and stored in a moist, shaded area protected from direct sunlight until the sowing time, three days after seed collection. The experiments were conducted during the planting season of 2021/2022 at the Center for Applied Research on the Environment and Sustainability, The American University in Cairo, Egypt (30°01′11.7″ N, 31°29′59.8″ E). The experiment was carried out in two greenhouses of different settings, i.e., a controlled fan and pad cooling greenhouse (GH) and a net house (NH) as illustrated in Figure 8. A soil planting mix was prepared by mixing 50% clay with 50% sand. Seeds of both species were planted in greenhouse plastic pots on 5 September 2021. Observations of young plant development were conducted over the following eight months, with physiological data recorded until the seedlings reached the transplantation stage.

The study followed a randomized complete block design (RCBD) of two main variables, i.e., planting under a controlled greenhouse and a net house for both mangrove species. Each main variable consisted of 4 treatments and 21 replicates, i.e., irrigation with 100% freshwater at 0.4%o (T1), irrigation with 100% saline water at 35%o (T2), 50% saline water and 50% freshwater at 18%o (T3), and brine water at 60%o (T4). The controlled cooling greenhouse conditions were characterized by light radiation 100–150 μmol m^−2^s^−1^, temperature 25 ± 5 °C during the study period and 15 ± 5 °C at night. The black-covered net house was exposed to the outside prevailing weather conditions as summarized in Table 3. Before sowing, the seeds were soaked in freshwater for 24 h and then sown at 5 cm depth in a potting mix with one plant per pot for both species. The saline irrigation water used in the experiment was collected from the Red Sea over time and the freshwater from the municipal water. The soil properties of the used soil in the experiment were as follows: Ph at 1:2.5 ratio was 8.25, EC (ds/m) at 2.52, Nitrogen 62 mg/kg, Phosphorous 7.8 mg/kg, Potassium 155 mg/kg. The soil particle distribution was also as follows: sand 55.5%, clay 40%, silt 4.5%, and organic matter 0.8%.

### 3.2. Data Collection

Agronomical plant data were collected every two weeks, starting from October 2021 till the end of May 2022. The measured parameters included germination percentage, seedling height (cm), stem diameter (cm), length of leaf blade (cm), leaf blade width (cm), and number of surviving leaves. The germination parameter was measured by observing, monitoring, and counting the germinated and surviving seedlings throughout the experimentation. The seedling height was measured by a calibrated meter ruler from the uniform soil surface to the topmost part of the seedling. The stem diameter was measured at a common stem spot for each seedling using a digital Vernier Caliper from Mitutoyo Incorp. The length of the leaf blade and blade width were measured using a calibrated meter ruler whereas the number of surviving leaves was measured by observation and counting.

### 3.3. Mineral Element Analysis

Plant samples were digested in an acid solution with a Berghof microwave digestion system (speedwave Entry DAP-60K). A sample weight of 300 mg was placed into the digestion vessel. To it, we added 8.0 mL of HNO_3_ at 65% with a power level of 90%. The mixture was shaken carefully and stirred with a clean glass bar. The vessel was closed 10 min after shaking. Then, the sample was heated in the microwave with the following program. Three steps were performed, the first step with a ramp time of 10 min, at 170 °C and a holding time of 5 min. The second step ran with a ramp time of 15 min, at 200 °C and a holding time of 3 min. The last phase ran with a ramp time of 10 min, 75 °C and a holding time of 1 min.

The resulting sample after cooling was transferred for element analysis. All measurements were performed using an Agilent 4210 MP-AES from Agilent, Santa Clara, CA, USA fitted with a double-pass cyclonic spray chamber and a OneNeb Series 2 nebulizer. Nitrogen was supplied using an Agilent 4107 Nitrogen Generator from Agilent too. All wavelengths were selected from the MP Expert software library, according to the sensitivity that was required. MP-AES operating conditions were as follows: read time of 3 s, pump speed of 15 rpm, uptake time of 15 s, and stabilization time of 25 s with a linear calibration fit.

### 3.4. Data Analysis

All data collected were analyzed using IBM-SPSS Statistical Tool (Version 22) and expressed as Mean ± SE. These data were subjected to a Leven’s test before analysis of variance (ANOVA) was conducted. The normality of data was checked using the Shapiro–Wilk test, and Q-Q plots test. *t*-test and ANOVA (both one and two-way ANOVA) were performed to detect significant differences in all the measured parameters, and the difference in means was analyzed by Duncan multiple range test (DMT) at α = 0.05.

## 4. Conclusions

In conclusion, Egypt is suffering from climate change impacts represented by sea-level rise, water scarcity, drought waves, the subsequent decline in agricultural productivity for many species, and other impacts. Mangroves are one of the key nature-based solutions that address climate change and its impacts. However, their survival and growth, mainly at the seedlings stage, are significantly affected by climatic and environmental factors, including temperature, relative humidity, solar radiation, salinity, and precipitation. Optimal seedling establishment often occurs within a narrow temperature range, as highly extreme temperatures can desiccate young plants, while very low temperatures may hinder physiological processes. Variations in the different climatic conditions are critical determinants of mangrove seedling resilience and distribution. Nevertheless, mangroves naturally grow in saline conditions, which makes them an excellent solution for countries suffering from drought and water scarcity as a nature-based solution for climate change. The study thus shows that *R. mucronata* prefers cooler temperatures for the establishment of seedlings, which is not the case with *A. marina*. This can also be related to their types of seeds, i.e., viviparous seeds with extended hypocotyl for *R. mucronata* and the cryptoviviparous seeds of *A. marina* where the latter is characterized by epigeal germination [36,37]. Also, according to our results, it can be concluded that mangrove species can optimally germinate and grow in both freshwater and 50% saline water; however, salinities above these levels would significantly inhibit and reduce seedling growth and establishment. On the other hand, it is scientifically proven that high ion concentrations in mangrove plant tissues indicate their ability to tolerate and adapt to high salinity conditions. This physiological trait reflects their capacity to offset increased salinities and maintain water uptake under varying salinity levels. The mangrove plants achieve this through different mechanisms such as ion compartmentalization, salt exclusion at the root level, and salt excretion via specialized glands. The study also revealed that mangroves cannot germinate and sustain growth while grown in brine water of 60%o salinity or higher seawater salinities caused by the negative impacts of climate change. Effects such as global warming and low levels of precipitation along the coastal areas may lead to an increased water salinity, which would be detrimental to naturally growing seeds or nurseries established on the seashore.

## Figures and Tables

**Table 1:** Stem diameters of Avicennia marina and Rhizophora mucronata grown in either a greenhouse or net house.

Treatment	Greenhouse Stem Diameter (cm) for *A. marina*								
	**Sep**	**Oct**	**Nov**	**Dec**	**Jan**	**Feb**	**Mar**	**Apr**	**May**
**T1**	0.00	0.00	0.00	0.45 ± 1.11	1.025 ± 1.59	0.85 ± 1.45	0.90 ± 1.52	1.88 ± 1.16	2.30 ± 1.34
**T2**	0.00	0.00	0.62 ± 1.15	1.28 ± 1.38	0.73 ± 1.73	0.00	0.00	0.00	0.00
**T3**	0.00	0.00	2.20 ± 1.58	2.96 ± 0.41	2.95 ± 0.39	1.88 ± 1.30	1.64 ± 1.60	1.18 ± 1.49	1.16 ± 1.51
**T4**	0.00	0.00	0.00	0.00	0.00	0.00	0.00	0.00	0.00
	**Net House Stem Diameter (cm) for *A. marina***								
**T1**	0.00	0.00	2.81 ± 0.31	2.79 ± 0.35	2.84 ± 0.40	2.79 ± 0.44	2.89 ± 0.25	2.92 ± 0.49	3.24 ± 0.31
**T2**	0.00	0.00	0.00	0.00	0.00	0.00	0.00	0.00	0.00
**T3**	0.00	0.00	2.68 ± 0.14	2.74 ± 0.13	1.92 ± 1.38	1.07 ±1.35	1.29 ± 1.66	0.36 ± 0.94	0.00
**T4**	0.00	0.00	0.00	0.00	0.00	0.00	0.00	0.00	0.00
**Treatment**	**Greenhouse Stem Diameter (cm) for *R. mucronata***								
	**Sep**	**Oct**	**Nov**	**Dec**	**Jan**	**Feb**	**Mar**	**Apr**	**May**
**T1**	10.6 ± 0.69	10.6 ± 0.69	10.6 ± 0.69	10.7 ± 0.67	10.94 ±1.17	10.6 ± 0.80	11.1 ± 1.27	11.14 ± 1.01	11.08 ± 1.13
**T2**	10.7 ± 0.72	10.7 ± 0.72	10.7 ± 0.72	10.37 ± 0.97	10.64 ± 1.11	10.35 ± 0.88	9.92 ± 1.21	9.87 ± 1.20	9.87 ± 1.27
**T3**	11.84 ± 0.77	11.84 ± 0.77	11.84 ± 0.77	11.14 ± 0.87	11.52 ± 0.75	11.21 ± 0.74	10.3 ± 0.62	10.11 ± 0.80	10.14 ± 1.09
**T4**	12.01 ± 2.62	12.01 ±2.62	12.01 ± 2.62	11 ± 1.49	11.36 1.16	11.36 ± 1.16	9.69 ± 1.29	9.69 ± 1.29	9.69 ± 1.29
	**Net House Stem Diameter (cm) for *R. mucronata***								
**T1**	9.89 ± 0.89	9.89 ± 0.89	9.89 ± 0.89	10.21 ± 1.04	10.94 ± 1.14	10.27 ± 1.65	10.04 ± 1.58	9.38 ± 2.24	8.93 ± 1.54
**T2**	7.22 ± 1.00	7.22 ± 1.00	7.22 ± 1.00	7.22 ± 1.00	7.22 ± 1.00	7.22 ± 1.00	7.22 ± 1.00	7.22 ± 1.00	7.22 ± 1.00
**T3**	9.06 ± 1.18	9.06 ± 1.18	9.54 ± 1.72	9.06 ± 1.18	9.06 ± 1.18	9.06 ± 1.18	9.06 ± 1.18	9.06 ± 1.18	9.06 ± 1.18
**T4**	9.2 ± 1.11	9.2 ± 1.11	9.2 ± 1.11	9.2 ± 1.11	9.2 ± 1.11	9.2 ± 1.11	9.2 ± 1.11	9.2 ± 1.11	9.2 ± 1.11

Note: Stem diameters are presented as mean ± standard deviation. T1: freshwater, T2: seawater, T3: mixed water, T4: brine.

**Table 2:** Mineral element composition of Avicennia marina and Rhizophora mucronata in g/kg concentration.

			Treatment	Na	Ca	Mg	K				Treatment	Na	Ca	Mg	K
***A.*** ***marina***	GH	Leaves	T1	38.861	7.8463	0.576	0.711	***R.*** ***mucronata***	GH	Leaves	T1	63.551	15.445	0.958	0.975
			T2	47.952	3.462	0.185	0.448				T2	96.653	256.32	2.160	1.145
			T3	39.569	4.492	0.335	0.461				T3	27.586	145.95	0.729	0.586
			T4	62.856	6.238	0.1543	0.4856				T4	80.315	358.82	2.396	3.948
		Roots	T1	39.368	3.8944	1.398	0.617			Roots	T1	38.833	172.25	0.936	0.954
			T2	58.849	3.850	0.226	0.419				T2	46.794	157.06	0.805	0.779
			T3	53.229	3.704	0.188	0.435				T3	42.223	135.16	0.729	0.778
			T4	73.259	2.360	0.107	0.124				T4	51.682	36.945	0.819	0.105
	NH	Leaves	T1	72.312	36.0	0.153	0.388		NH	Leaves	T1	70.852	48.776	0.765	0.761
			T2	58.893	71.56	0.2785	0.631				T2	27.324	150.78	1.204	0.771
			T3	73.760	63.943	0.226	0.887				T3	49.677	304.33	3.884	1.044
			T4	68.763	162.7	0.632	1.089				T4	83.492	97.839	0.532	0.918
		Roots	T1	68.194	60.774	0.855	0.632			Roots	T1	58.584	203.27	1.117	0.828
			T2	52.881	91.846	0.367	0.864				T2	68.913	96.072	0.522	0.801
			T3	74.214	105.43	0.322	1.051				T3	65.849	29.47	0.793	0.655
			T4	67.575	153.91	0.877	1.121				T4	70.103	41.858	0.783	0.558

**Table 3:** Atmospheric weather data during the experimental period.

Months	Rainfall (mm)	Min. Air Temp (°C)	Max. Air Temp (°C)	Min. Relative Humidity (%)	Max. Relative Humidity (%)	Solar (MJ/m^2^)
Sept	0.00	25.92	31.70	72.46	85.16	26.403
Oct	0.06	22.74	28.23	76.12	84.43	22.962
Nov	0.07	20.07	25.28	75.34	86.45	19.947
Dec	0.16	14.04	18.09	70.44	81.56	13.668
Jan	0.25	10.88	15.06	68.19	81.79	10.658
Feb	0.43	12.50	17.27	72.73	83.31	12.546
Mar	0.00	13.45	18.51	64.97	78.81	13.875
Apr	0.01	22.04	29.33	54.03	69.31	22.576
May	0.00	22.98	29.42	55.43	73.55	24.572

## Data Availability

All data presented in this study are available in this article; requests to access the raw data can be made through the corresponding author.
